# The Role of Phosphatidylinositol-3-Kinase and AMP-Activated Kinase in the Rapid Estrogenic Attenuation of Cannabinoid-Induced Changes in Energy Homeostasis

**DOI:** 10.3390/ph4040630

**Published:** 2011-04-12

**Authors:** Garrett S. Jeffery, Kelly C. Peng, Edward J. Wagner

**Affiliations:** Department of Basic Medical Sciences, Western University of Health Sciences, Pomona, CA 91766, USA

**Keywords:** estrogen, POMC, appetite, cannabinoids, PI3K, AMPK

## Abstract

We sought to determine the involvement of phosphatidyl inositol 3-kinase (PI3K) and AMP-activated protein kinase (AMPK) in the estrogenic antagonism of the cannabinoid regulation of energy homeostasis. Food intake and body weight were evaluated in ovariectomized female guinea pigs treated s.c. with estradiol benzoate (EB) or its sesame oil vehicle, or the CB_1_ receptor antagonist AM251 or its cremephor/ethanol/0.9% saline vehicle. AMPK catalytic subunit, PI3K p85α regulatory subunit and proopiomelanocortin (POMC) gene expression was assessed via quantitative RT-PCR in microdissected hypothalamic tissue. Whole-cell patch clamp recordings were performed in hypothalamic slices. Both EB and AM251 decreased food intake and weight gain, and increased AMPKα1, AMPKα2 and PI3K p85α gene expression in the mediobasal hypothalamus. 17β-Estradiol rapidly and markedly attenuated the decreases in glutamatergic miniature excitatory postsynaptic current (mEPSC) frequency caused by the cannabinoid receptor agonist WIN 55,212-2 in POMC neurons. This rapid estrogenic diminution of cannabinoid-induced decreases in mEPSC frequency was blocked by the estrogen receptor (ER) antagonist ICI 182,780 and the PI3K inhibitor PI 828, the latter of which also prevented the AM251-induced increase in mEPSC frequency. In addition, the AMPK activator metformin reversed the EB-induced decreases in food intake and weight gain and restored the ability of WIN 55,212-2 to reduce mEPSC frequency. These data reveal that estrogens physiologically antagonize cannabinoid-induced changes in appetite and POMC neuronal activity by activating PI3K and inhibiting AMPK. As such, they provide insight into the neuroanatomical substrates and signal transduction mechanisms upon which these counter-regulatory factors converge in the control of energy homeostasis.

## 1. Introduction

Ovarian estrogens are essential for the control of homeostasis. For example, they regulate the reproductive axis via negative and positive feedback that allows for the proper timing of ovulation, and estrogen priming of the reproductive tract maximizes the likelihood of blastocyst implantation into the uterine endometrium [1,2]. Estrogens also lower body temperature [3-5] and reduce appetite [5-9] in several different animal models, and in human females periovulatory peak levels of these steroids produce the same effects [10]. In addition, estrogens increase dopamine release in both the striatum and nucleus accumbens of the basal forebrain, and by doing so they can positively modulate sexual behavior, drug abuse and the extrapyramidal control of motor function [11-14].

It has long been known that estrogens exert many of their hormonal effects by activating intracellular receptors like estrogen receptor (ER)α and ERβ that ultimately bind to estrogen-responsive elements located upstream of the coding regions of target genes to influence their transcription. However, it is becoming widely accepted that they can also alter cell function on a much more rapid time scale than that accounted for by gene transcription alone [15]. Some of these rapid estrogenic actions are mediated via ERα tethered to the plasma membrane through palmitoylation and interactions with caveolin proteins [16-18]. For example, ERα introduced to striatal neurons through a viral vector is confined largely to the plasma membrane, and enhances rapid estrogenic influences on amphetamine-induced rotational behavior and K^+^-evoked γ-aminobutyric acid (GABA) release [19]. ERα also interacts with metabotropic glutamate receptors in a caveolin-dependent manner to modulate L-type calcium channel activity and phosphorylation of cAMP response element-binding protein in rat hippocampus, as well as in the limbic-hypothalamic circuit that controls rat sexual behavior [18,20,21]. On the other hand, estrogen also rapidly diminishes the coupling of G_i/o_-coupled receptors to their inwardly-rectifying K^+^ (GIRK) channels in anorexigenic, glucose-responsive guinea pig proopiomelanocortin (POMC) neurons by activating a phospholipase C (PLC)/protein kinase C (PKC)/protein kinase A (PKA) pathway that does not involve stimulation of ERα, ERβ, ER-X or GPR30 [15,22-24].

POMC neurons also express phosphatidylinositol-3-kinase (PI3K), which converts phosphatidylinositol_4,5_-bisphosphate (PIP2) into phosphatidylinositol_3,4,5_-triphosphate (PIP3) that positively modulates K_ATP_ channel activity and cell excitability [25]. PI3K in POMC neurons is activated by the humoral factors leptin and insulin via during a positive energy balance [25-27], and estrogens upregulate the expression of the regulatory subunit of this enzyme in the arcuate nucleus (ARC) of the guinea pig [28-30]. In addition, the cellular energy sensing AMP-activated protein kinase (AMPK) regulates the glucose responsiveness of POMC neurons [26], and mediates the hyperphagic effects of ghrelin and endogenous cannabinoids [31,32]. Moreover, the hypothalamic expression of both endogenous cannabinoids and the AMPK α_2_ subunit is suppressed by anorexigenic leptin [33,34], and given that estrogens and leptin share a common signal transduction pathway [27] the same may hold true for these steroids. Of course, both PI3K and AMPK are expressed in other hypothalamic neuronal populations, including agouti-related protein (AgRP)-containing neurons known to co-express neuropeptide Y (NPY) and γ-aminobutyric acid (GABA), and impinge upon POMC neurons to inhibit their electrical activity [26,35-37].

We have shown previously that estrogens exert a rapid and sustained attenuation of the cannabinoid regulation of energy homeostasis as evidenced by their ability to disrupt cannabinoid receptor agonist-induced hyperphagia, hypothermia and neurotransmission at POMC synapses [38,39]. As such, we tested the hypothesis that estrogens can act via a membrane ER to alter the expression and activity of AMPK and PI3K and thereby rapidly antagonize cannabinoid-induced presynaptic inhibition of glutamate input onto POMC neurons and thus hyperphagia. To this end, we compared the respective abilities of estradiol benzoate (EB) and the cannabinoid CB1 receptor antagonist AM251 to reduce food intake, weight gain, as well as AMPK and PI3K gene expression in the ARC and hypothalamic ventromedial nucleus (VMN). We also evaluated the ability of the ER antagonist ICI 182, 780, the PI3K inhibitor PI 828 and the AMPK activator metformin to block the estrogenic diminution of cannabinoid signaling at POMC synapses and, in some cases, the EB-mediated hypophagia. Finally, we ascertained whether PI3K blockade could preclude the facilitation of excitatory neurotransmission at POMC synapses caused by CB1 receptor antagonism with AM251.

## 2. Experimental

### 2.1. Animals

All animal procedures described in this study are in accordance with institutional guidelines based on NIH standards. Female Topeka guinea pigs (350-650 g) were obtained from Elm Hill Breeding Labs (Chelmsford, MA, USA) or bred in-house, kept under controlled temperature (69-73 °F) and light (12 h on: 12 h off), and provided with food and water *ad libitum*. They were ovariectomized under ketamine/xylazine anesthesia (33 mg/kg and 6 mg/kg, respectively; s.c.) at least 4 days prior to experimentation.

### 2.2. Drug

All compounds were purchased from Tocris Cookson, Inc. (Ellisville, MO, USA) unless otherwise indicated. For the feeding studies, EB (Steraloids, Newport, RI, USA) was initially prepared as a 1 mg/mL stock solution in punctilious ethanol. A known quantity of this stock solution was added to a volume of sesame oil sufficient to produce a final concentration of 100 μg/mL following evaporation of the ethanol. *N*-(Piperidin-1-yl)-5-(4-iodophenyl)-1-(2,4-dichlorophenyl)-4-methyl-1*H*-pyrazole-3-carboxamide (AM251) and *N,N*-dimethylimidodicarbonimidic diamide hydrochloride (metformin) were dissolved in cremephor/ethanol/0.9% saline (1/1/18; v/v/v) and 0.9% saline, respectively. For the electrophysiological experiments, tetrodotoxin (TTX) with citrate, 6-imino-3-(4-methoxyphenyl)-1(6H)-pyridazinebutanoic acid hydrobromide (SR 95531) and metformin were dissolved in Ultrapure H_2_O to stock concentrations of 1 mM, 10 mM and 50 mM, respectively. (*R*)-(+)-[2,3-Dihydro-5-methyl-3-(4-morpholinylmethyl)pyrrolo[1,2,3-de]-1,4-benzoxazin-6-yl]-1-naphtha lenylmethanone mesylate (WIN-55,212-2), 2-(4-morpholinyl)-8-(4-aminopheny)l-4H-1-benzopyran-4-one (PI 828) and AM251 were each dissolved in dimethyl sulfoxide to a stock concentration of 10 mM. 17β-Estradiol (E_2_) and 7a,17b-[9-[(4,4,5,5,5-pentafluoropentyl)sulfinyl]nonyl] estra-1,3,5(10)-triene-3,17-diol (ICI 182,780) was dissolved in punctilious ethanol to stock concentrations of 2.7 and 1 mM, respectively.

### 2.3. Feeding Behavior

The feeding studies were performed as previously described [40,41]. Briefly, food intake was monitored around the clock for seven days using a Comprehensive Lab Animal Monitoring System (Columbus Instruments; Columbus, OH, USA). The AMPK activator metformin (50 mg/kg; s.c.), the CB_1_ receptor antagonist AM251 (3 mg/kg; s.c.), or their 0.9% saline and cremephor/ethanol saline (1/1/18; v/v/v) vehicles (1 mL/kg; s.c.) were injected each morning at 8:00 a.m. EB (10 μg; s.c.) or its sesame oil vehicle (0.1 mL; s.c.) were given every other morning.

### 2.4. Quantitative PCR

At the end of the seven-day monitoring period the animals were decapitated and the brain rapidly removed. Three coronal slices (1 mm in thickness) spanning the rostral-caudal extent of the ARC and VMN were prepared using a guinea pig brain matrix (Ted Pella, Inc.; Redding, CA, USA), and stored in RNAlater (Ambion, Inc.; Austin, TX, USA) for 2-3 hr. The ARC and VMN were then microdissected from 2-3 of these slices according to the guinea pig brain atlas generated by Tindal [42].

All primer sequences were synthesized by and purchased from Invitrogen (Carlsbad, CA, USA), and were designed to cross at least one intron-exon boundary with the aid of Clone Manager 8.0 software (Sci-Ed Software, Cary, NC, USA). Guinea pig specific primers for the AMPK α1 and AMPK α2 catalytic subunits were designed by looking for regions of high homology between the human, rat and mouse gene sequences. The resultant primer set for AMPKα1 (forward: 5′-TTGCGTGTACGAAGGAAG-3′, base pairs 1335-1353; reverse: 5′-GAGTAGCAGTCCCTGATTTG-3′, base pairs 1479-1461) produced an amplicon 145 base pairs in length that spanned exons 8 and 9 of AMPKα1 gene, whereas that for AMPKα2 (forward: 5′-GTCTGCTGTGGATTACTG-3′, base pairs 443-460; reverse: 5′-CAATCTGCCTGAGA TGAC-3′, base pairs 638-620) yielded an amplicon 196 base pairs in length that spanned exons 4-6. Primers for POMC, preproNPY and the p85α regulatory subunit for PI3K were obtained by analyzing sequences originally designed, characterized and used elsewhere [28,30] that also ultimately met in our hands the efficiency and melting point dissociation criteria described below.

Total RNA from each sample was extracted using the RNAqueous-Micro Kit (Ambion, Inc.) as per the manufacturer's specifications. It was then quantified with a NanoVue spectrophotometer (GE Healthcare Life Sciences, Piscataway, NJ, USA), and treated with DNase I (DNA free, Ambion; 37 °C for 30 min) to minimize contamination by genomic DNA. SuperScript™ III reverse transcriptase (200 U; Invitrogen), along with 3 μL 5X buffer, 15 mM MgCl_2_, 10 mM dNTP, 100 ng random hexamer primers, 40 U/μL RNaseOUT™ and 100 mM dithiothreitol (in diethylpyrocarbonate water) were used to generate cDNA from 200 ng RNA (20 μL total reaction volume). Reverse transcription was carried out as follows: 5 min at 25 °C, 60 min at 50 °C, 15 min at 70 °C and 5 min at 4 °C. The resultant cDNA was then diluted 20× with Nuclease-free water (Ambion Inc.) and stored at -20 °C.

For quantitative PCR, cDNA (3-4 μL) was amplified with a Power SYBR® Green master mix (Applied Biosystems, Carlsbad, CA, USA) using an ABI 7300 Fast Real-time PCR machine. Standard curves for each pair of primers were generated using serial dilutions of mediobasal hypothalamic cDNA in triplicate to calculate their efficiency (E) via the following relationship: E = 10(^-1/m^)-1, where ‘m’ equals the slope of the standard curve. All of the primer efficiencies were greater than 90%. The amplification of cDNA started with an initial denaturation at 95 °C for 10 min, followed by 45 cycles of amplification at 94 °C for 15 sec (denaturing) and at 60 °C for 30 sec (annealing), and completed with a dissociation step for melting point analysis consisting of 35 cycles of 95 °C for 15 sec, 60 °C to 95 °C at 1 °C increments over 1 min and 95 °C for 15 sec. All amplification runs included the appropriate positive and negative controls as used by others, and relative quantification was performed using the comparative CT method as described in detail elsewhere [28-30].

### 2.5. Electrophysiology

Electrophysiological recordings from ARC neurons were performed using an *in vitro* hypothalamic slice preparation as previously described [39,40]. Briefly, electrode resistances varied from 3-8 MΩ. Membrane currents were recorded in voltage clamp with access resistances ranging from 8-22 MΩ, and underwent analog-digital conversion via a Digidata 1322A interface coupled to pClamp 8.2 software (Axon Instruments). The access resistance, as well as the resting membrane potential and the input resistance, were monitored throughout the course of the recording. If the access resistance deviated greater than 20% of its original value, the recording was ended. To ascertain the extent of the rapid estrogenic attenuation of cannabinoid receptor agonist-induced decreases in glutamatergic mEPSCs, cells were perfused in artificial cerebrospinal fluid in the presence of 500 nM TTX and 10 μM SR 95531 to block GABA_A_ receptor-mediated synaptic input, and also with 100 nM E_2_ or its ethanol vehicle (0.00376% by volume), for 10-15 minutes. In some experiments designed to determine if the estrogenic modulation of cannabinoid signaling at ARC synapses is ER-, PI3K- and/or AMPK-mediated, either the ER antagonist ICI 182,780 (1 μM), the PI3K inhibitor PI 828 (10 μM) or the AMPK activator metformin (500 μM) was co-administered along with E_2_. Baseline recordings were performed from a holding potential of -75 mV for 3-4 minutes. Slices were then perfused with the cannabinoid receptor agonist WIN 55,212-2 (1 μM) for 3-4 minutes, and 3-4 more minutes of data were collected in the presence of the agonist. In other experiments designed to ascertain whether pharmacologic blockade of CB1 receptor-mediated signaling at ARC synapses is PI3K-mediated, slices were pre-treated with PI 828 or vehicle for 10-15 min, subjected to baseline intracellular recording for 3-4 min, perfused with AM251 (1 μM) for 3-4 min, followed by the collection of 3-4 min worth of additional data in the presence of the antagonist. Measurements were obtained from at least 100 contiguous mEPSCs and were analyzed to determine alterations in frequency and amplitude prior to, and in the presence of, these compounds. After recording, some slices were processed for immunohistofluorescence as described previously [43].

### 2.6. Statistics

Comparisons between two groups were made with either the Student's t-test, the Kolmogorov-Smirnov test, or the Mann-Whitney W test. Comparisons between multiple treatment groups were performed using either the Kruskal-Wallis test followed by analysis of the median-notched, Box-and-Whisker plot, or the one-way or two-way analysis of variance (ANOVA) followed by the Least Significant Difference (LSD) test. Differences were considered statistically significant if the probability of error was less than 5%.

## 3. Results

### 3.1. Experiment #1: The Effects of EB and CB1 Receptor Blockade on Food Intake and Weight Gain

**Figure 1:**
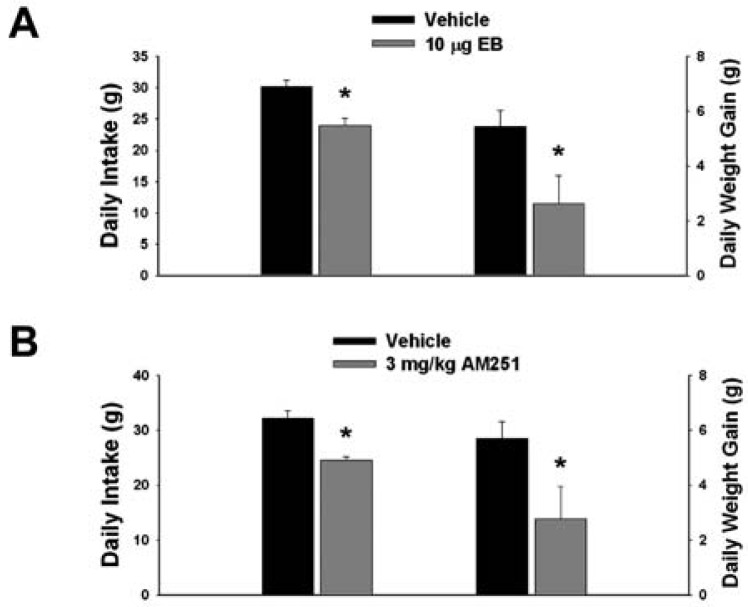
EB (**A**) and AM251 (**B**) decrease food intake and body weight. Bars represent means and vertical lines 1 SEM of the daily food intake and weight gain measured every day for seven days. *, Values measured in EB- and AM251-treated animals that are significantly different (Student's t-test; p < 0.05; n = 5-8) than those measured in vehicle-treated controls.

Our first objective was to compare the effects of EB (10 μg; s.c.) and the CB1 receptor antagonist AM251 (3 mg/kg; s.c.) on food intake and weight gain. As shown in Figure 1A, EB reduced by ∼20% the mean cumulative intake measured over the course of 24 hr (vehicle: 30.16 ± 0.99 g/day; EB: 23.96 ± 1.15 g/day; p < 0.001) and slowed by ∼50% the average rate of weight gained over the same period (vehicle: 5.44 ± 0.58 g/day; EB: 2.63 ± 1.02 g/day; p < 0.03). Nearly identical effects were found with AM251. As shown in Figure 1B, AM251 evoked a ∼25% decrease in daily food intake (vehicle: 32.14 ± 1.39 g/day; AM251: 24.48 ± 0.64 g/day; p < 0.001) and a ∼50% decrease in daily weight gain (vehicle: 5.69 ± 0.62 g/day; AM251: 2.76 ± 1.18 g/day; p < 0.04).

### 3.2. Experiment #2: The Effects of EB and CB1 Receptor Blockade on PI3K and AMPK Gene Expression in the Mediobasal Hypothalamus

**Figure 2:**
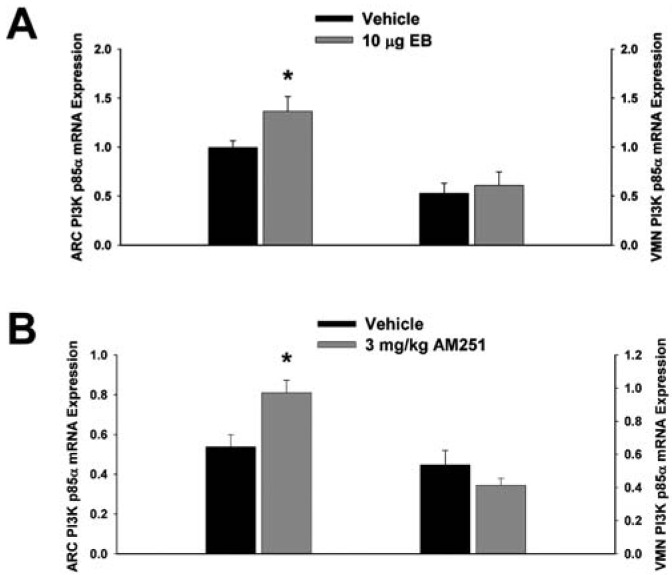
Both EB and AM251 increase PI3K expression in the ARC but not the VMN. Bars represent means and vertical lines 1 SEM of the PI3K, p85α regulatory subunit mRNA expression determined in ARC and VMN tissue harvested after the feeding experiments. *, Values measured in EB- and AM251-treated animals that are significantly different (Student's t-test; p < 0.05; n = 5-8) than those measured in vehicle-treated controls.

**Figure 3:**
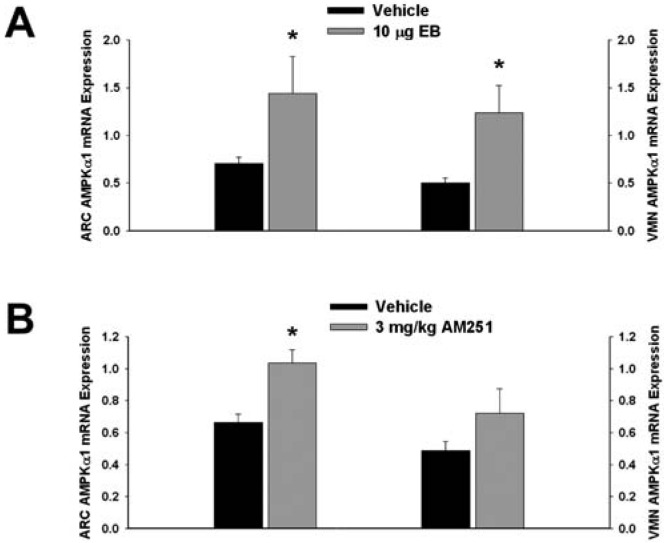
Both EB and AM251 increase AMPKα1 expression in the ARC and VMN. Bars represent means and vertical lines 1 SEM of the AMPKα1 catalytic subunit mRNA expression determined in ARC and VMN tissue harvested after the feeding experiments. *, Values measured in EB- and AM251-treated animals that are significantly different (Student's t-test; p < 0.05; n = 5-8) than those measured in vehicle-treated controls.

**Figure 4:**
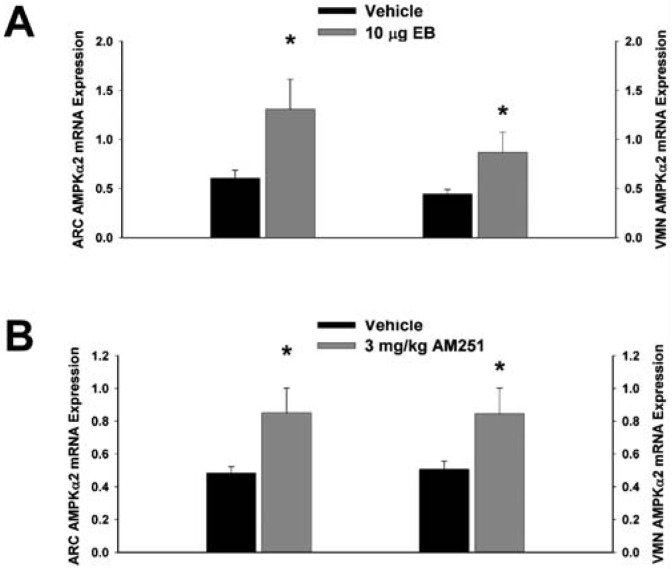
Both EB and AM251 increase AMPKα2 expression in the ARC and VMN. Bars represent means and vertical lines 1 SEM of the AMPKα2 catalytic subunit mRNA expression determined in ARC and VMN tissue harvested after the feeding experiments. *, Values measured in EB- and AM251-treated animals that are significantly different (Student's t-test; p < 0.05; n = 5-8) than those measured in vehicle-treated controls.

Next we examined whether EB and AM251 modulate the expression of PI3K and AMPK in the ARC and VMN; two peptides highly relevant to the control of energy balance in the mediobasal hypothalamus. Both EB (Figure 2A) and AM251 (Figure 2B) elevated the expression of the p85α regulatory subunit of PI3K in the ARC (p < 0.04 and p < 0.02, respectively) but not the VMN (p < 0.64 and p < 0.26, respectively). As with all of the qPCR primers used in the present study, the melting point dissociation plots for p85α and the housekeeping reference gene glyceraldehyde-3-phosphate dehydrogenase (GAPDH) exhibited single peaks and were highly efficient (92.5% and 91%, respectively). Therefore, we feel confident the observed changes truly reflect the effects of estrogens and CB1 receptor antagonism on the expression of these genes. EB (Figure 3A) also increased the expression of the AMPKα1 catalytic subunit by over 2X in both the ARC (p < 0.05) and VMN (p < 0.02), while the CB1 receptor antagonist (Figure 3B) increased AMPKα1 expression in the ARC (p < 0.01) and caused a modest, albeit statistically insignificant increase in the VMN (p < 0.13). These striking similarities are further substantiated by the fact that both EB and AM251 increased expression of the AMPKα2 catalytic subunit in both the ARC (Figure 4A; p < 0.02 and p < 0.01, respectively) and VMN (Figure 4B; p < 0.04 and p < 0.04, respectively).

### 3.3. Experiment #3: The Effects of PI 828 on the ER-Sensitive Cannabinoid Agonist-induced Decrease, and the CB1 Receptor Antagonist-induced Increase, in Excitatory Synaptic Input onto POMC Neurons

**Figure 5:**
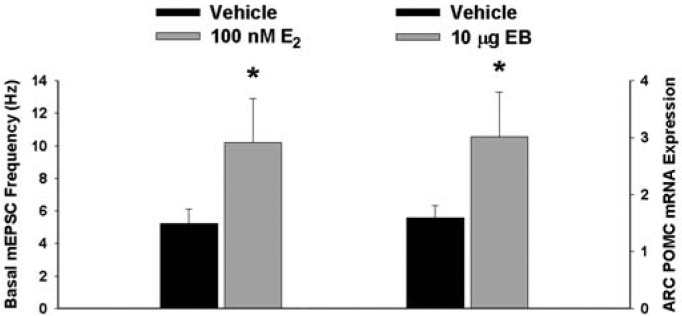
Estrogens increase basal mEPSC frequency and POMC expression. Bars represent means and vertical lines 1 SEM of the basal mEPSC frequency and POMC mRNA expression in the ARC. *, Values measured in E_2_-treated slices or EB-treated animals that are significantly different (Student's t-test; p < 0.05; n = 5-8) than those measured in vehicle-treated controls.

**Figure 6:**
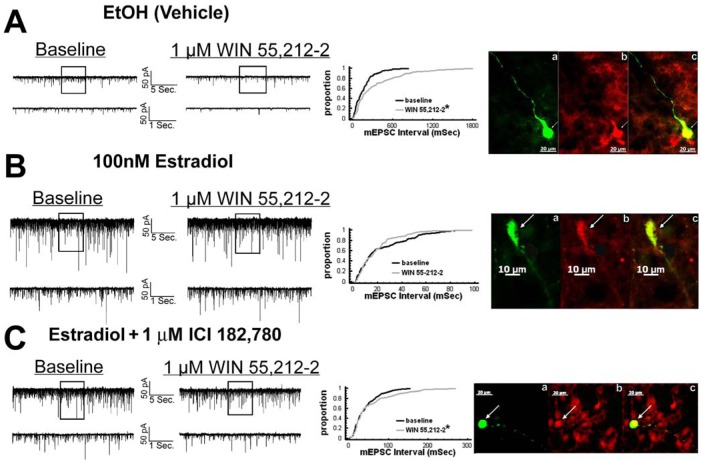
E_2_ rapidly attenuates the cannabinoid receptor agonist-induced presynaptic inhibition of glutamate release onto POMC neurons via an ER receptor-mediated mechanism. **A**, Spontaneous mEPSCs recorded in a vehicle-treated arcuate neuron at baseline (left) and following exposure to 1 μM WIN 55,212-2 (right). To the right of the membrane current traces is the cumulative probability plot illustrating the increase in interval (inverse of frequency) between contiguous mEPSCs in the cell on the left. To the right of the cumulative probability plot is the double-labeling of this same neuron that is immunopositive for β-endorphin (**a**, biocytin-streptavidin-AF488 labeling; **b**, β-endorphin immunofluorescence visualized by AF546; **c**, composite overlay). **B**, Spontaneous mEPSCs in a cell perfused with 100 nM E_2_ at baseline (left) and following exposure to 1 μM WIN 55,212-2 (right). To the right of the membrane current traces is the cumulative probability plot illustrating the interval between contiguous mEPSCs that substantiates the lack of cannabinoid effect in the E_2_-treated slice containing the cell on the left. To the right of the cumulative probability plot is the double-labeling of this same neuron that is immunopositive for CART (**a**, biocytin-streptavidin-AF488 labeling; **b**, CART immunofluorescence visualized by AF546; **c**, composite overlay). **C**, Spontaneous mEPSCs recorded in a neuron co-treated with 100 nM E_2_ and 1 μM ICI 182,780 at baseline (left) and following exposure to 1 μM WIN 55,212-2 (right). To the right of the membrane current traces is the cumulative probability plot illustrating the ability of ICI 182,780 to restore the cannabinoid receptor agonist-induced decrease in mEPSC frequency. To the right of the cumulative probability plot is the double-labeling of this same neuron that is immunopositive for α-MSH (**a**, biocytin-streptavidin-AF488 labeling; **b**, α-MSH immunofluorescence visualized by AF546; **c**, composite overlay). *, Distribution of the mEPSC interval in the presence of WIN 55,212-2 that is significantly different (Kolmogorov-Smirnov test, p < 0.05) than that observed under basal conditions.

**Figure 7:**
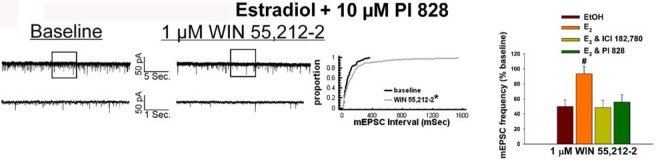
The estrogenic attenuation of the cannabinoid-induced decrease in glutamate release in blocked by the PI3K inhibitor PI 828. Spontaneous mEPSCs in a cell perfused with E_2_ and 10 μM PI 828 at baseline (left) and following exposure to 1 μM WIN 55,212-2 (right). To the right of the membrane current traces is the cumulative probability plot illustrating the interval between contiguous mEPSCs that substantiates the ability of PI 828 to restore the inhibitory effect of WIN 55,212-2 on mEPSC frequency. To the right of the cumulative probability plot is the composite bar graph illustrating the efficacy of ICI 182,780 and PI 828 to block the E_2_-induced attenuation of the decrease in mEPSC frequency caused by WIN 55,212-2. Bars represent means and vertical lines 1 SEM of the cannabinoid receptor agonist-induced decrease in mEPSC frequency. *, Distribution of the mEPSC interval in the presence of WIN 55,212-2 that is significantly different (Kolmogorov-Smirnov test, p < 0.05) than that observed under basal conditions. #, Values measured in E_2_-treated slices that are significantly different (Kruskal-Wallis/median-notched Box-and-Whisker analysis; p < 0.05; n = 5-8) than those measured in the other treatment conditions.

We have shown previously that estrogens rapidly antagonize cannabinoid-induced hyperphagia and hypothermia, which can be attributed, at least in part, to their ability to exert a rapid and sustained negative modulation of inhibitory, presynaptic cannabinoid influences on glutamatergic neurotransmission at anorexigenic POMC synapses [38-40,44]. This powerful estrogenic attenuation of cannabinoid-induced presynaptic inhibition of glutamatergic neurotransmission at POMC synapses most likely contributes to the E_2_-induced ∼2X increase in the basal mEPSC frequency (vehicle: 5.22 ± 0.89 Hz; E_2_: 10.22 ± 2.70 Hz; p < 0.05; Figure 5) but not amplitude (vehicle: -11.9 ± 1.4 pA; E_2_: -12.6 ± 0.6 pA; p < 0.62; not shown), and POMC gene expression in the ARC (p < 0.03; Figure 5; see also the baseline mEPSC traces from vehicle- and E_2_-treated slices in (Figure 6A and Figure 6B). We first wanted to determine if this estrogenic disruption of the cannabinoid signaling at POMC synapses occurs via an ER receptor-mediated mechanism. The ability of E_2_ to negate the dramatic increase in mEPSC interval, and thus the decrease in mEPSC frequency, caused by the cannabinoid receptor agonist WIN 55,212-2 (Figure 6A and 6B) was rendered completely ineffective in the presence of the ER antagonist ICI 182,780 (1 μM; Figure 6C), which restored the ability of WIN 55,212-2 to increase mEPSC interval (and decrease mEPSC frequency) to the same extent as in cells from vehicle-treated slices (Figure 6C and 7). Of the 32 neurons (out of 84 total) evaluated immunohistochemically for *post-hoc* phenotypic indentification, 28 were immunopositive for β-endorphin, cocaine- and amphetamine-regulated transcript (CART) or α-melanocyte-stimulating hormone (α-MSH) like those shown in Figure 6.

**Figure 8:**
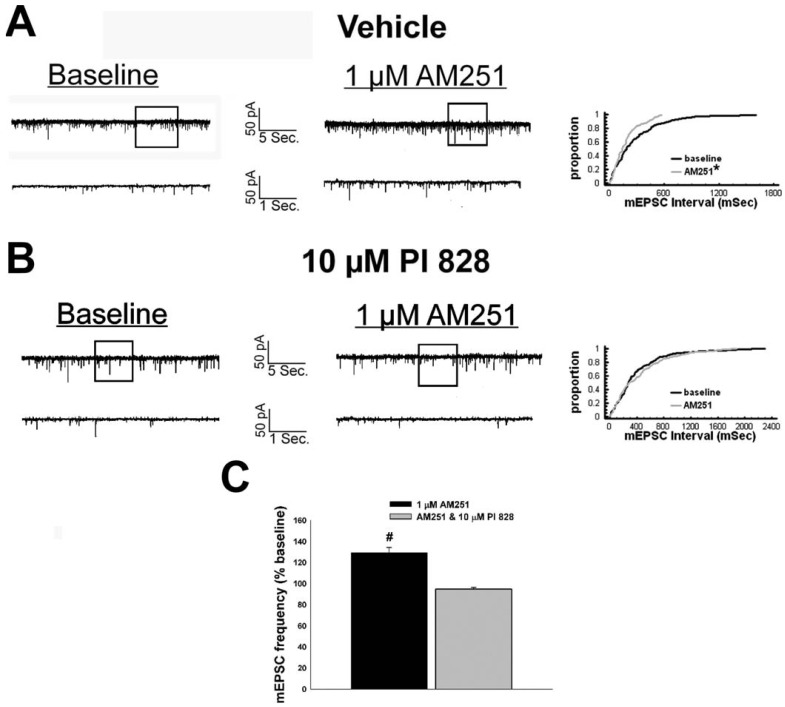
PI3K inhibition with PI 828 prevents the AM251-induced increase in glutamatergic input onto POMC neurons. **A**, Spontaneous mEPSCs recorded in a vehicle-treated arcuate neuron at baseline (left) and following exposure to 1 μM AM251 (right). To the right of the membrane current traces is the cumulative probability plot illustrating the decrease in interval between contiguous mEPSCs in the cell on the left. **B**, Spontaneous mEPSCs in a cell perfused with 10 μM PI 828 at baseline (left) and following exposure to 1 μM AM251 (right). To the right of the membrane current traces is the cumulative probability plot illustrating the interval between contiguous mEPSCs that demonstrates the lack of antagonist effect in the PI 828-treated slice containing the cell on the left. **C**, This composite bar graph illustrates the efficacy of PI 828 to block the AM251-induced increase in mEPSC frequency. Bars represent means and vertical lines 1 SEM of the mEPSC frequency in the presence of PI 828 and/or AM251 normalized to baseline control conditions. *, Distribution of the mEPSC interval in the presence of AM251 that is significantly different (Kolmogorov-Smirnov test, p < 0.05) than that observed under basal conditions. #, AM251-induced changes in mEPSC frequency measured cells from vehicle-treated slices that are significantly different (Mann-Whitney W test; p < 0.05; n = 4-5) than those measured in PI 828-treated slices.

Given that estrogens and AM251 upregulate PI3K in the ARC (see Figure 2), we also wanted to ascertain if this signaling molecule is involved in the ER receptor-mediated hindrance of the cannabinoid agonist-induced suppression, and the AM251-induced facilitation, of glutamatergic input at POMC synapses. As such, we pre-treated slices with the 110 KDa, PI3K catalytic subunit inhibitor PI 828 (10 μM), alone and in conjunction with E_2_. As can be surmised from the membrane current traces, cumulative distribution plot and composite bar graphs in Figure 7, PI 828 markedly diminished the effect of the steroid at POMC synapses; thereby enabling WIN 55,212-2 to decrease mEPSC frequency to levels that very closely approximate those observed in cells from vehicle-treated slices. In addition, while PI 828 *per se* was without effect on basal mEPSC frequency (vehicle: 4.3 ± 0.9 Hz; PI 828: 4.6 ± 1.6 Hz; p < 0.90), it completely blocked the AM251-induced increase in mEPSC frequency (Figure 8).

### 3.4. Experiment #4: The Effects of Metformin on Estrogenic Alterations in Food Intake, Weight Gain and Cannabinoid Agonist-induced Inhibition of Excitatory Neurotransmission at POMC Synapses

**Figure 9:**
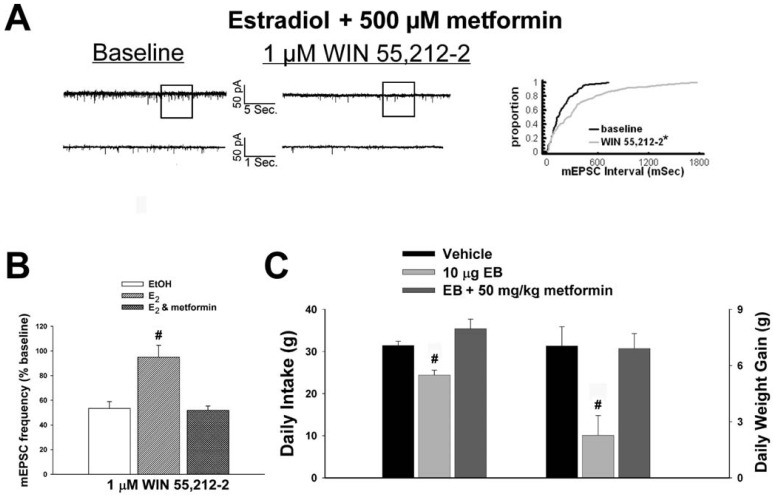
AMPK activation with metformin reverses the estrogen-induced disruption of cannabinoid signaling at POMC synapses, as well as the decreases in food intake and weight gain. **A**, Spontaneous mEPSCs recorded in a neuron co-administered 100 nM E_2_ and 500 μM metformin at baseline (left) and following exposure to 1 μM WIN 55,212-2 (right). To the right of the membrane current traces is the cumulative probability plot illustrating the ability of metformin to restore the cannabinoid receptor agonist-induced decrease in mEPSC frequency. **B**, Composite bar graph illustrating the effectiveness of metformin to negate the E_2_-induced diminution of the decrease in mEPSC frequency caused by WIN 55,212-2. Bars represent means and vertical lines 1 SEM of the cannabinoid receptor agonist-induced decrease in mEPSC frequency. **C**, Metformin blocks the estrogen-induced decrease in food intake and weight gain. Bars represent means and vertical lines 1 SEM of the daily food intake and weight gain measured every day for seven days. *, Distribution of the mEPSC interval in the presence of WIN 55,212-2 that is significantly different (Kolmogorov-Smirnov test, p < .05) than that observed under basal conditions. #, Values measured in E_2_-treated slices or EB-treated animals that are significantly different (**B**: Kruskal-Wallis/median-notched Box-and-Whisker analysis; p < .05; n = 5-8; **C**: one-way ANOVA/LSD; p < 0.05; n = 4-7) than those measured under the other treatment conditions.

As mentioned above, we found that EB stimulated the expression of the AMPKα1 and α2 catalytic subunits in the mediobasal hypothalamus (see Figure 3A and 4A). However, leptin, which lowers energy intake and has many signal transduction features in common with estrogens, acutely reduces the hypothalamic expression of AMPKα2 [34]. To investigate if AMPK is involved in the estrogenic impediment of cannabinoid-induced changes in energy homeostasis, or whether its upregulation is merely a compensatory response to seven days of EB exposure, we assessed the effects of the AMPK activator metformin on estrogen-mediated changes in food intake, weight gain and cannabinoid signaling at POMC synapses. Metformin (500 uM) rendered the steroid ineffective in antagonizing the cannabinoid-induced decrease in mEPSC frequency (Figure 9A and 9B), and systemic administration (50 mg/kg; s.c.) reversed EB-induced decreases in daily food intake and weight gain (Figure 9C).

### 3.5. Experiment #5: The Effects of EB and CB1 Receptor Blockade on NPY Gene Expression in the ARC

**Figure 10:**
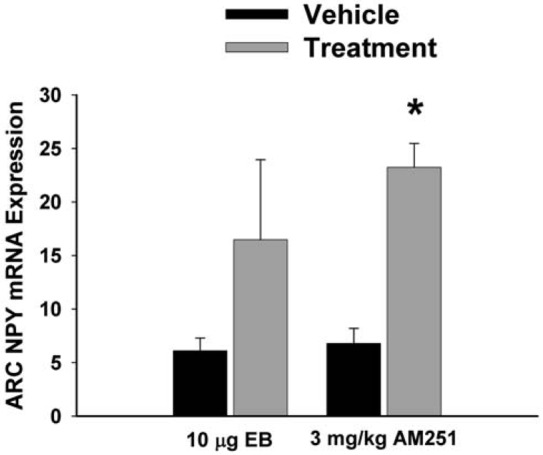
The effects of EB and AM251 on NPY expression in the ARC. Bars represent means and vertical lines 1 SEM of the preproNPY mRNA expression determined in ARC tissue harvested after the feeding experiments. *, Values measured in EB- and AM251-treated animals that are significantly different (Student's t-test; p < 0.05; n=5-8) than those measured in vehicle-treated controls.

The ability of the AMPK activator metformin to acutely reverse EB-induced reductions in food intake and weight gain, as well as the estrogenic attenuation of cannabinoid agonist-induced alterations in excitatory neurotransmission at POMC synapses, suggests that the EB- and AM251-induced upregulation of AMPKα1 and AMPKα2 occurs ultimately as a compensatory response in the face of the negative energy balance created by these anorexigenic stimuli. It is known that under fasting conditions constitutively active AMPK appreciably augments hypothalamic expression of orexigenic NPY, AgRP and melanin-concentrating hormone (MCH[34]). Therefore, we wanted to determine if the EB- and AM251-induced decreases in food intake and weight gain also were associated with alterations in ARC preproNPY gene expression. As shown in Figure 10, ARC preproNPY expression was elevated 2-3X in EB- and AM251-treated animals (p <0.15 & p <0.0001, respectively)

## 4. Discussion

Taken together, these data demonstrate that estrogens rapidly antagonize the cannabinoid regulation of energy homeostasis via an ER receptor-mediated mechanism that stimulates PI3K activity and suppresses AMPK activity in the mediobasal hypothalamus. These conclusions are based on the following observations: 1) EB, like the CB1 receptor antagonist AM251, decreased food intake and weight gain, and increased PI3K gene expression in the ARC; 2) E_2_ rapidly attenuated the cannabinoid receptor agonist-induced decrease in mEPSC frequency at anorexigenic POMC synapses, which was prevented by the ER antagonist ICI 182,780 and the PI3K inhibitor PI 828, and associated with increased glutamatergic tone and POMC gene expression; 3) PI 828 completely blocked AM251-induced increase in glutamate release onto POMC neurons; and 4) while EB and AM251 increased AMPK gene expression in the ARC and VMN, concomitant AMPK activation with metformin actually reversed the steroid-induced decrease in consumption and weight gain, and restored the ability of cannabinoids to presynaptically glutamatergic input onto POMC neurons.

### 4.1. Estrogens Antagonize the Cannabinoid System and Thereby Promote a Negative Energy Balance

The estrogen-induced decrease in food consumption observed presently is consistent with that reported in previous rodent studies [6-8]. In human females, energy intake is lowest during the late follicular phase of the ovulatory cycle—near the time of ovulation—when estrogen levels are at their peak and unopposed by progesterone [10]. ERα undoubtedly plays a role in the estrogenic reduction in ingestive behavior—as evidenced by the fact that the ERα agonist PPT decreases food intake and meal size in ovariectomized rats [45], and ERα silencing in the VMN results in obesity associated with metabolic syndrome [46]. On the other hand the estrogenic ligand STX, which does not bind ERα, ERβ, ER-X or GPR30 [15,23,24], also decreases food intake concomitant with reductions in meal frequency and size [5]; indicating that a G_q_-coupled membrane estrogen receptor also imparts a significant contribution in this regard.

The estrogen-induced changes in food intake, weight gain, and the expression of AMPK and PI3K in the mediobasal hypothalamus, were strikingly similar to those observed with the CB1 receptor antagonist AM251. The CB1 receptor antagonist Rimonabant was on the market in Europe as an anti-obesity drug, and was considered quite efficacious in lowering weight, reducing waist circumference and ameliorating the dyslipidemia associated with the metabolic syndrome [47,48]. This, coupled with the observation that ERα silencing in the VWN promotes the metabolic syndrome [46], would indicate that estrogens physiologically antagonize the endogenous cannabinoid system in the ventral diencephalon. Indeed, we have demonstrated previously that estrogens powerfully diminish cannabinoid-induced hyperphagia and hypothermia, as well as the augmentation of an A-type K^+^ current in anorexigenic ARC POMC neurons [39]. This is in accordance with the recent work of Riebe and co-workers [49], in which they report that a 10 μg dose of EB—the same amount we used in the present study—decreased cannabinoid receptor binding in hypothalami from ovariectomized female rats by ∼50%.

### 4.2. Estrogens Disrupt Presynaptic Cannabinoid Signaling Upstream of POMC Neurons in Part via an ER-mediated Mechanism that Stimulates PI3K Activity

Estrogens also rapidly and markedly attenuate the ability of cannabinoid receptor agonists to inhibit glutamate release at POMC synapses. We observed this effect within 20 minutes from the time E_2_ exposure was initiated—well below the six hours necessary to see increased excitatory, asymmetric synapses being formed with POMC perikarya [9]. In the present study we also show that this latter effect is blocked by both ER and PI3K antagonists. This indicates that estrogens act via an ER receptor-mediated mechanism to rapidly enhance PI3K activity, which then uncouples presynaptic CB1 receptors from their effector system(s) to disrupt the inhibition of glutamate release at POMC synapses. This would increase glutamatergic tone onto POMC neurons. Indeed, we saw that estrogens rapidly increased basal mEPSC frequency in these cells, which is consistent with the estrogen-induced decrease in the paired-pulse ratio observed in developing VMN neurons [50]. This estrogen-induced diminution of cannabinoid signaling and resultant disinhibition of POMC neurons may also facilitate the increased POMC expression that we observed presently, and others have shown previously [28,30,51].

The question arises: how might this be accomplished? It is well-established that in POMC neurons themselves estrogens rapidly impede postsynaptic G_i/o_-coupled receptors from GIRK channels by activating a PLC/PKC/PKA pathway [15,23,24] that is also important in mediating rapid estrogenic responses in hippocampal neurons and the limbic-hypothalamic circuit that regulates sexual behavior in females [20,52]. More recently, it has been shown that estrogens can also activate PI3K to functionally uncouple GABA_B_ receptors from GIRK channels in POMC neurons [29]. This activation can be quite rapid—as evidenced by the fact that in NG108-15 neurons estrogens stimulate the phosphorylation of Akt within 30 minutes in a PI3K-sensitive fashion [53]. Both PLC and PI3K are similar in their ability to deplete membrane PIP_2_ reserves—PLC converts PIP_2_ into diacylglycerol (which activates PKC) and inositol 1,4,5-triphosphate, whereas PI3K converts PIP_2_ into PIP_3_. PIP_2_ is a positive allosteric modulator of GIRK channels—as evidenced by the fact that dialyzing POMC neurons with PIP_2_ prevents GABA_B_/GIRK uncoupling in these cells [57].

It could be that estrogenic activation of these pathways ultimately elicits the synthesis and release of retrograde messengers from POMC neurons that disrupt the coupling of presynaptic CB1 receptors from their effector system(s). In support of this idea is the observation that corticosteroids exert rapid negative feedback on the stress axis by causing the release of endogenous cannabinoids from parvocellular neurons in the hypothalamic paraventricular nucleus (PVN) that act transynaptically to inhibit glutamate release [55,56]. In addition membrane associated ERα activates endothelial nitric oxide synthase (eNOS) in a PI3K/Akt-dependent manner [16,57], and in the hypothalamus estrogens increase neuronal NOS (nNOS) activity via stimulation of the PI3K/Akt pathway [58,59]. Moreover, estrogen potentiates L-arginine-induced stimulation of gonadotropin-releasing hormone (GnRH) secretion from hypothalamic explants via a mechanism involving increases in intracellular calcium and the activation of PKA, PKC, mitogen-activated protein kinase and nitric oxide synthesis that served to augment the glutamatergic stimulation of GnRH secretion [60]. The nitric oxide thus formed could act as a retrograde messenger as has been shown in the hippocampus with long-term potentiation [61]. Along these lines, glucocorticoids rapidly facilitate GABA release at magnocellular synapses in the PVN by eliciting the synthesis and release of nitric oxide, which acts in a retrograde fashion to stimulate neurotransmitter secretion from GABAergic nerve terminals [62]. Future studies will explore the intriguing possibility that estrogens impair the cannabinoid-induced presynaptic inhibition of glutamate release at POMC synapses by promoting the production of retrograde messengers in POMC neurons.

### 4.3. Estrogens Disrupt Cannabinoid-induced Changes in Energy Balance in Part by Inhibiting AMPK Activity in the Mediobasal Hypothalamus

In addition to reducing food intake and weight gain, we found that both EB and AM251 increased AMPK gene expression in the mediobasal hypothalamus. Estrogens have been reported to rapidly activate Akt and AMPK in rat soleus [63], which is similar to the stimulatory effect of leptin on AMPK activity in skeletal muscle [32]. Other metabotropic G_q_-coupled receptors activated by ghrelin, histamine and platelet-activating factor also stimulate AMPK activity in endothelial cells by a mechanism that apparently involves concomitant activation of PI3k/Akt and eNOS [64,65]. By contrast, leptin acutely decreases AMPK expression in the both the ARC and PVN, and constitutively active and dominant negative AMPK expressed in mice via an adenovirus vector respectively increase and decrease food intake and weight gain [34]. In addition, endogenous cannabinoids mediate the ghrelin-induced hyperphagia and upregulation of hypothalamic AMPK activity [31]. AMPK also is essential for the glucose responsiveness of POMC neurons [22,26,66]—as evidenced by the fact that the glucoprivation-induced decrease in the firing frequency of these cells is abolished in POMC-AMPKα2 knockout mice [26]. Moreover, we found that concomitant AMPK activation with metformin *in vivo* reversed the estrogen-induced reduction in food intake and weight gain, while *in vitro* it prevented the estrogenic attenuation of the cannabinoid-induced presynaptic inhibition of glutamate release at POMC synapses. Thus, our results would indicate that estrogens acutely decrease AMPK activity, and that the upregulation of AMPK gene expression caused by EB and AM251 serves as an attempt to offset the negative energy balance created by the anorexigenic stimuli.

Given the fact that the mediobasal hypothalamus is a heterogeneous region containing many different cell types, it is quite possible that this compensatory, estrogen-induced increase in AMPK expression is conferred to neurons other than POMC neurons. While AMPK is expressed in POMC neurons, a considerable body of evidence suggests that most of its regulation of energy balance is due to actions on upstream elements of POMC synapses (e.g., NPY/AgRP neurons, glutamatergic neurons; [26,34,67]). Indeed, dominant negative AMPK reduces NPY and AgRP mRNA expression in the ARC, and AgRP-AMPKα2 knockout mice exhibit an age-dependent lean phenotype compared to their wildtype controls [26,34]. In addition, estrogens acutely blunt NPY-induced hyperphagia [68]. Moreover, constitutively active AMPK markedly enhances NPY, AgRP and MCH (but not POMC) gene expression under fasting conditions[34], which is in agreement with the 2-3X increase in ARC NPY expression that we observed in the present study with EB (and AM251) treatment over the course of the seven-day monitoring period. Collectively these observations are consistent with the notion that estrogens can rapidly target the upstream component(s) of POMC synapses to bring about increases in glutamatergic tone – a scenario similar to that described in developing VMN neurons [50].

The manner in which AMPK contributes to the estrogenic decrease in CB1 receptor/effector coupling at POMC synapses is uncertain. While estrogens can clearly attenuate the coupling of metabotropic G_i/o_-coupled receptors to postsynaptic GIRK channels via direct actions on POMC neurons [2], estrogens can also act upstream to modulate NPY-induced changes in the excitability of these cells [69]. Metabotropic G_q_-coupled receptor-mediated signaling generates contemporaneous increases in PI3K/Akt, eNOS and AMPK activity in endothelial cells [64,65], In the VMN insulin stimulates, whereas leptin and glucose inhibit, the activation of glucose-sensitive neurons via the PI3K/Akt, AMPK and nNOS pathway that is observed following the reduction in ambient glucose levels [70,71]. However, we found that EB and AM251 altered PI3K expression in the ARC but not the VMN, which may reflect another difference in the way these two hypothalamic regions respond to nutrient and hormonal signals. The possibility exists that the steroid- and cannabinoid-induced increases in AMPK expression truly reflect increases in activity, as we observed with PI3K. However, the estrogenic effects that we encountered in the presence of metformin would indicate otherwise. It could be that the estrogen-induced decrease in AMPK activity physiologically antagonizes the reported downstream CB1 receptor-mediated activation of AMPK [31], which may account for the reduction in CB1 receptor agonist potency and efficacy to decrease glutamate release in the presence of the steroid. Future studies are clearly needed to address these important scientific questions.

### 4.4. Summary

In conclusion, these results reveal that estrogens reduce appetite, weight gain and rapidly disinhibit POMC neurons via a physiologic antagonism of the endogenous cannabinoid system that involves, at least in part, an activation of PI3K and an inhibition of AMPK. These data impart insight into the neuroanatomical substrates and signaling mechanisms upon estrogens and cannabinoids converge in the control of energy homeostasis. Finally, our findings have implications for how therapeutic, cannabinoid-induced orexigenesis in women may vary over the course of the reproductive cycle.
